# Haglund Syndrome: A Mechanical Cause of Posterior Heel Pain

**DOI:** 10.7759/cureus.112434

**Published:** 2026-07-10

**Authors:** Houda Bennani, Yahya Elharras, Ali Haidar, Ittimade Nassar, Kaoutar Imrani

**Affiliations:** 1 Radiology Department, Ibn Sina University Hospital Center, Rabat, MAR; 2 Radiology Department, Ibn Sina University Hospital, Faculty of Medicine and Pharmacy, Mohammed V University, Rabat, MAR

**Keywords:** achilles tendinopathy, enthesitis, haglund deformity, heel pain, retrocalcaneal bursitis

## Abstract

Haglund syndrome has become an important consideration in the differential diagnosis of posterior heel pain. It results from mechanical conflict between a prominent posterosuperior calcaneal tuberosity, the Achilles tendon, and the surrounding bursae. We report the case of a 45-year-old woman presenting with chronic posterior heel pain associated with a visible deformity of the posterior ankle. Imaging demonstrated a prominent calcaneal tuberosity, Achilles tendinopathy, and mild retrocalcaneal bursitis. The clinical presentation and imaging findings are discussed, highlighting the role of imaging techniques in evaluating posterior heel pain, confirming the diagnosis of Haglund syndrome, and excluding other causes of posterior heel pain, including inflammatory enthesitis associated with spondyloarthropathies.

## Introduction

Posterior heel pain is a common clinical complaint that may result from a variety of osseous, tendinous, and inflammatory conditions affecting the hindfoot [1]. Among these, Haglund deformity refers to a prominent posterosuperior calcaneal tuberosity. First described by Patrick Haglund in 1927, this bony abnormality may cause repetitive mechanical conflict with the Achilles tendon insertion and the adjacent bursae, leading to pain and swelling. The resulting clinical condition is known as Haglund syndrome [2-4].

Patients with Haglund syndrome typically present with posterior heel pain, although the clinical presentation may overlap with several other conditions, including isolated insertional Achilles tendinopathy, retrocalcaneal bursitis, inflammatory enthesitis associated with spondyloarthropathies, and, less commonly, calcaneal stress fractures. Consequently, imaging plays a pivotal role in establishing the diagnosis and excluding alternative causes of posterior heel pain [1,5].

Conventional radiography is the first-line imaging modality for evaluating calcaneal morphology, excluding calcaneal stress fractures, and identifying the characteristic posterosuperior calcaneal prominence and thickening of the retrocalcaneal soft tissues. When symptoms persist or are associated with soft-tissue abnormalities, MRI provides a comprehensive assessment of the Achilles tendon, adjacent bursae, and bone marrow. It enables accurate characterization of associated lesions and helps confirm the diagnosis [1,5,6].

We report the case of a middle-aged woman presenting with chronic posterior heel pain due to Haglund syndrome. This case highlights the complementary roles of conventional radiography and MRI in establishing the diagnosis, assessing associated tendon abnormalities, and differentiating this condition from other causes of posterior heel pain.

## Case presentation

A 45-year-old woman presented with progressive posterior pain of the right ankle that had evolved over several months. She had no relevant medical history and reported no history of trauma. Although she did not participate in regular sports or recreational physical activity, she remained independently active in her daily life, performing household chores, shopping, and regular walking. She usually wore open-backed shoes and only occasionally wore closed-backed shoes, which exacerbated the pain and intermittently caused localized swelling in the retrocalcaneal region. She had been using an elastic ankle brace without clinical improvement.

**Figure 1:**
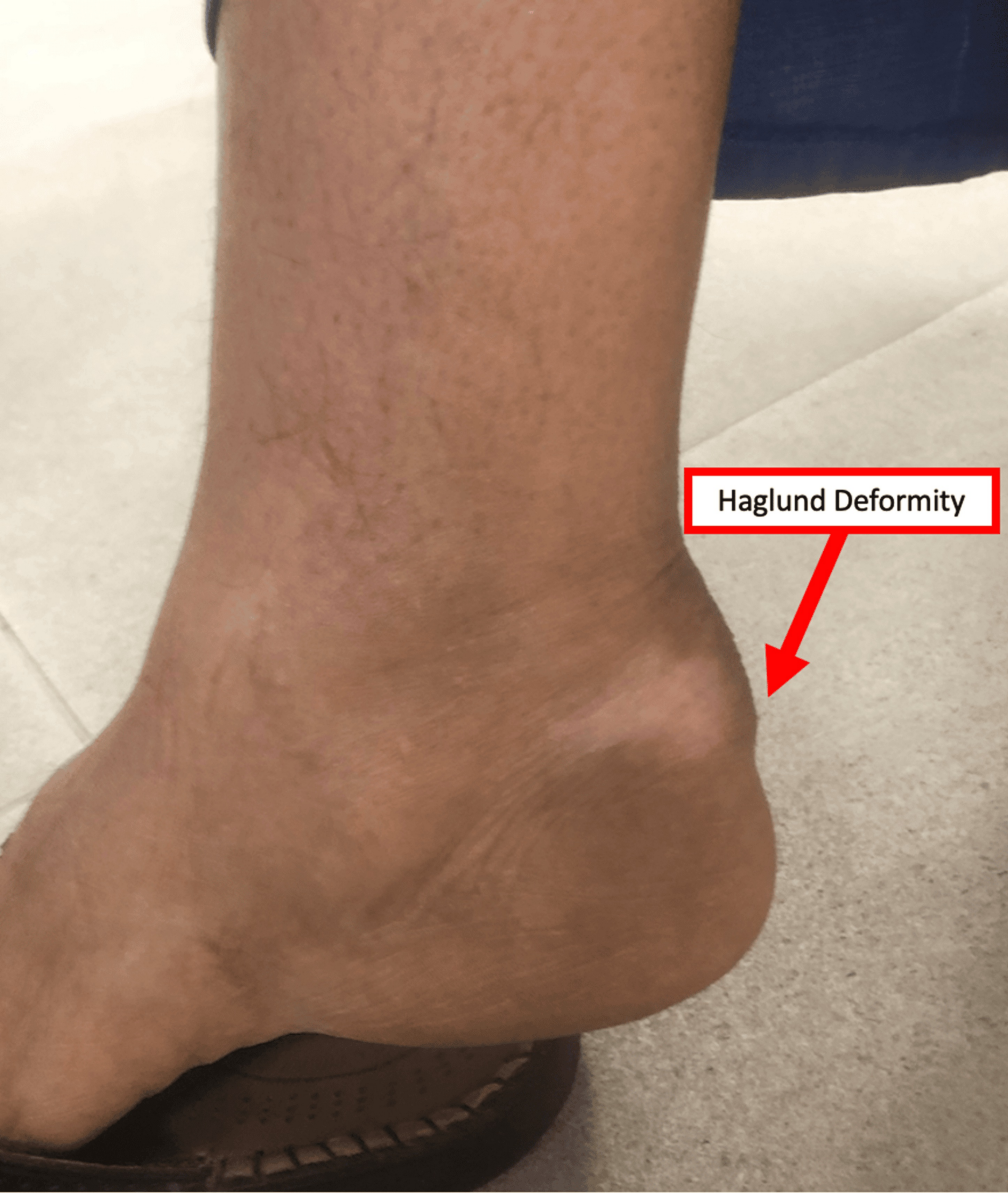
Visible Haglund deformity Clinical photograph of our case obtained during physical examination demonstrating a prominent posterosuperior heel deformity with localized discoloration at the retrocalcaneal region.

Clinical examination revealed a visible deformity of the posterior heel with localized skin discoloration on inspection, secondary to repetitive friction blisters, and a palpable bony prominence at the posterior aspect of the calcaneus near the Achilles tendon insertion (Figure 1). Active and passive ankle movements were painful, with a limited range of motion.

**Figure 2:**
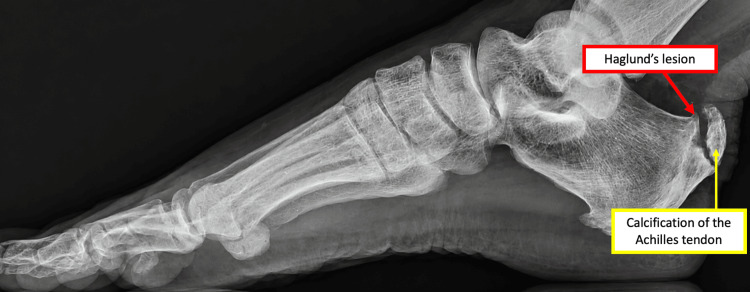
Radiographic features of Haglund syndrome Lateral foot radiograph demonstrating a prominent bony growth of the posterosuperior angle of the calcaneus and macrocalcification at the distal Achilles tendon insertion.

Conventional radiography of the right foot confirmed the presence of a bony prominence at the posterosuperior aspect of the calcaneus, together with a calcification at the distal Achilles tendon insertion (Figure 2).

**Figure 3:**
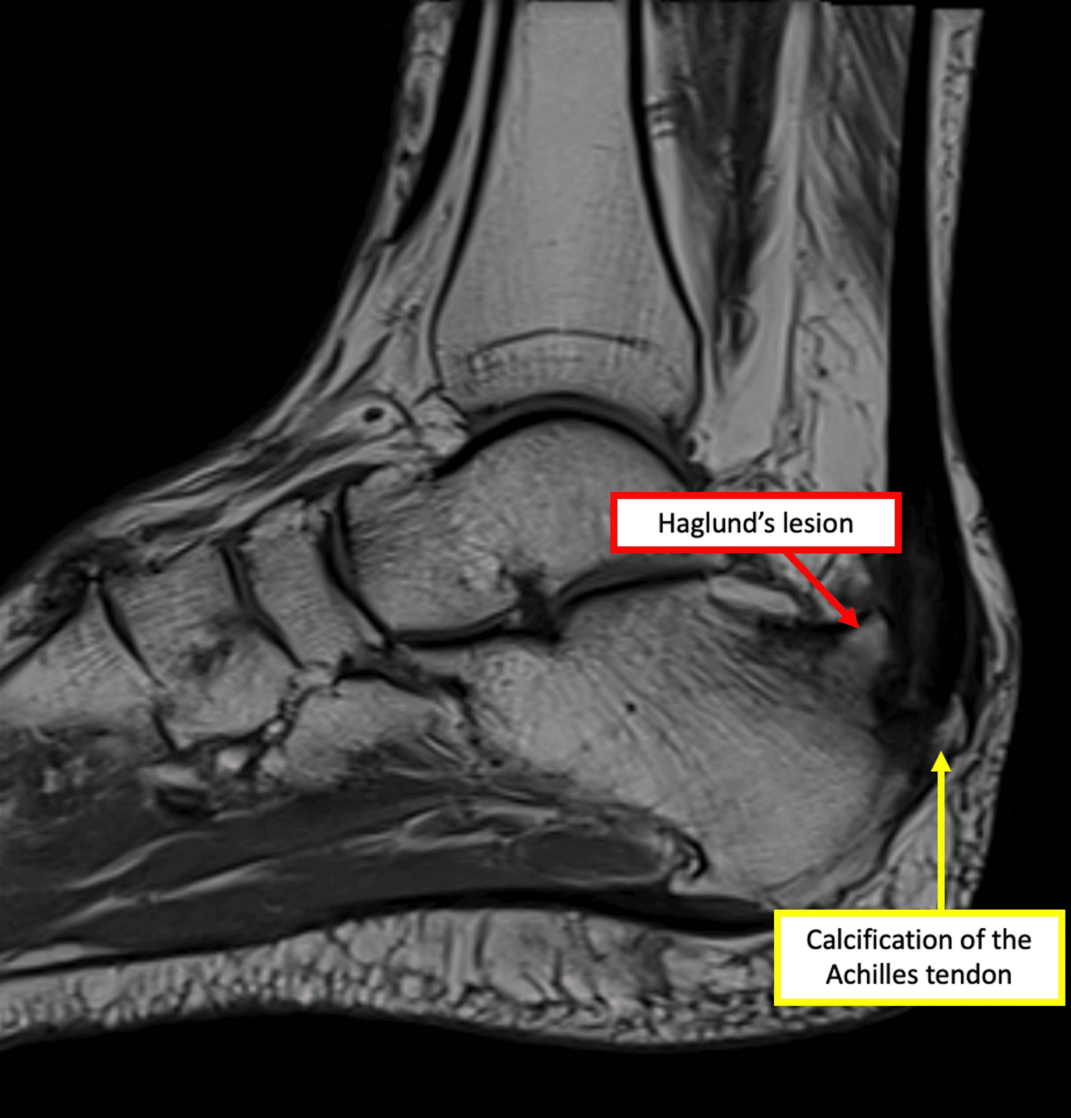
Calcaneal prominence and Achilles tendon insertion changes in Haglund syndrome on MRI Sagittal T1-weighted MRI sequence of the right ankle demonstrating prominent posterosuperior calcaneal tuberosity (Haglund deformity) associated with calcification of the Achilles tendon distal insertion. MRI: magnetic resonance imaging

**Figure 4:**
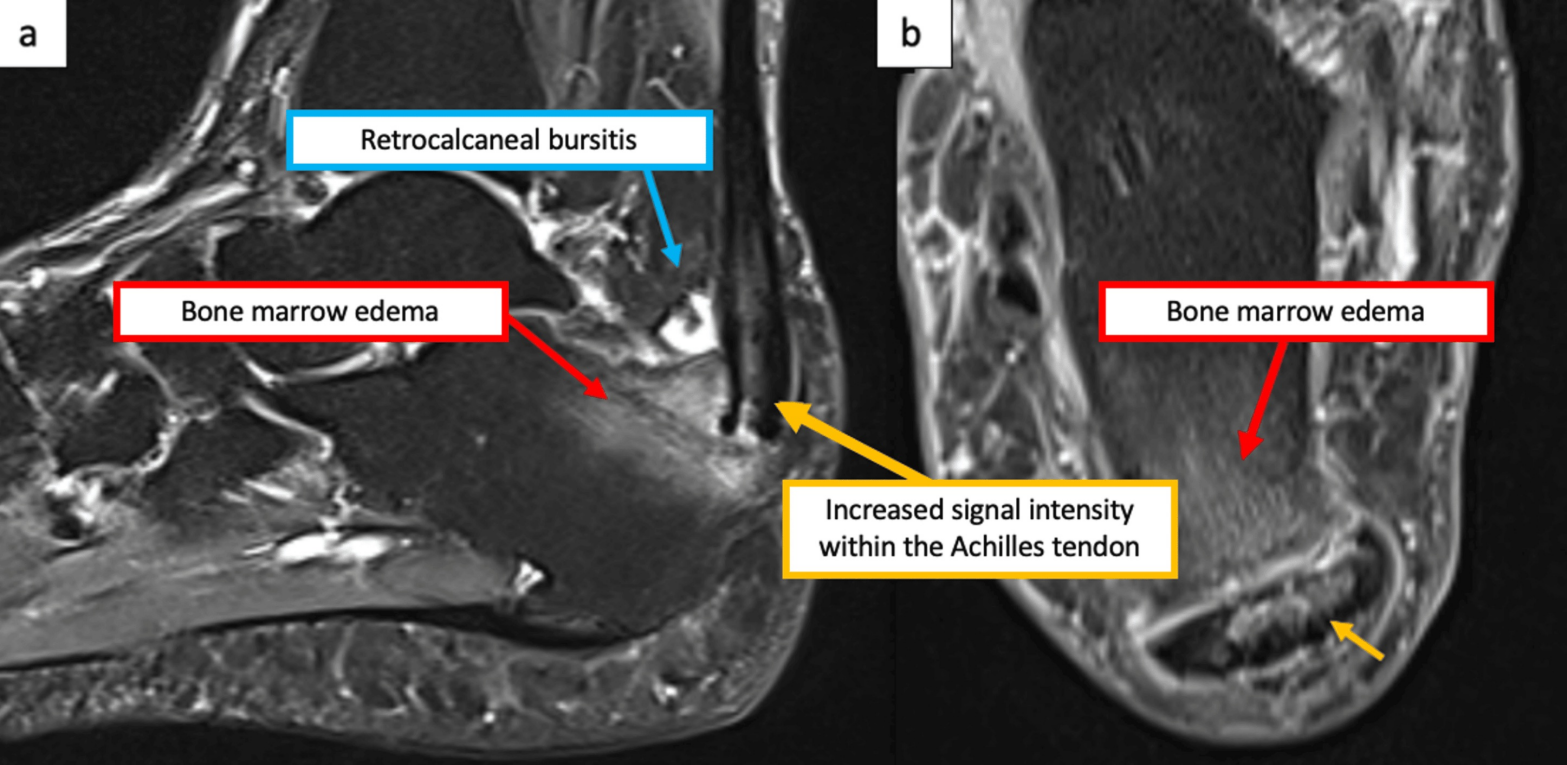
MRI features of calcaneo-Achilles impingement in Haglund syndrome Sagittal (a) and axial (b) proton density fat-suppressed MRI sequences demonstrating hyperintense signal within the posterosuperior calcaneus (red arrow), compatible with bone marrow edema; increased signal intensity at the Achilles tendon insertion (yellow arrow) suggestive of insertional tendinopathy; and minimal fluid signal within the retrocalcaneal bursa (blue arrow). MRI: magnetic resonance imaging

Ultrasonography was subsequently performed, revealing a thickening of the Achilles tendon at the retrocalcaneal region. Given the suspicion of Achilles tendinopathy, the patient was referred to our department for a complementary MRI. The MRI protocol included sagittal T1-weighted and proton density fat-suppressed sequences, as well as axial proton density fat-suppressed images. MRI confirmed the posterosuperior calcaneal prominence and calcification at the distal Achilles tendon insertion. MRI additionally demonstrated thickening of the Achilles tendon with increased signal intensity on fluid-sensitive sequences, consistent with insertional inflammatory and degenerative Achilles tendinopathy. A focal area of hyperintense signal within the posterosuperior calcaneus was also observed, compatible with bone marrow edema. Minimal fluid distension of the retrocalcaneal bursa and mild inflammatory changes in the surrounding peritendinous soft tissues were also identified. These imaging findings supported the diagnosis of Haglund syndrome (Figures 3-4).

Following the diagnosis, the patient was managed conservatively with shoe modifications to alter heel height, orthotic support, physiotherapy, and nonsteroidal anti-inflammatory medication. She reported partial improvement in pain following treatment, with no residual limitation of her daily activities.

## Discussion

The exact etiology of Haglund syndrome remains multifactorial and is thought to involve a combination of anatomical and biomechanical factors. The condition is primarily attributed to repetitive mechanical impingement between a prominent posterosuperior calcaneal tuberosity and the Achilles tendon insertion, leading to irritation of the surrounding soft tissues, degenerative changes in the Achilles tendon, and inflammation of the retrocalcaneal bursa [2,3]. Several contributing factors have been proposed, including morphological variations of the calcaneus, increased tension of the Achilles tendon, repetitive microtrauma, and external mechanical pressure from rigid or closed-back footwear, which together predispose patients to insertional Achilles tendinopathy [3,4,7]. Epidemiologically, Haglund syndrome is most commonly reported in adults and has been described more frequently in physically active individuals, with some studies suggesting a higher prevalence among women [1,4].

Clinically, the condition is characterized by posterior heel pain and localized swelling. The prominent posterosuperior aspect of the calcaneus is often referred to as Haglund deformity, Mulholland deformity, retrocalcaneal exostosis, or “pump bump” [4,8]. Symptoms are typically aggravated by physical activity and wearing rigid footwear.

**Figure 5:**
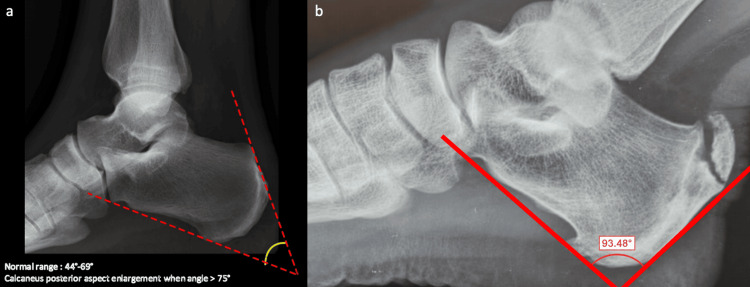
Radiographic evaluation of the Fowler-Philip angle Lateral radiographs illustrating measurement of the Fowler-Philip angle. (a) Reference diagram showing the technique used to measure the angle between the posterior and plantar cortices of the calcaneus (adapted from https://radiopaedia.org/cases/fowler-philip-angle licensed under Creative Commons Attribution-NonCommercial-ShareAlike 3.0 (CC BY-NC-SA 3.0)). (b) Application of the measurement in the present case demonstrating a markedly increased Fowler-Philip angle (93.5°).

Radiographic examination remains the first-line imaging modality for evaluating suspected Haglund deformity, as it allows visualization of the characteristic posterosuperior calcaneal prominence and enables assessment of calcaneal morphology using measurements such as the Fowler-Philip angle (Figure 5) [4,5].

CT may provide a more detailed evaluation of the osseous anatomy and can be useful in selected cases, particularly for better characterization of the calcaneal prominence or for preoperative planning. However, conventional radiography and CT do not adequately assess the associated soft-tissue abnormalities [4,5]. Ultrasound can be useful for detecting retrocalcaneal bursitis, typically appearing as fluid distension of the retrocalcaneal bursa, and for assessing Achilles tendon abnormalities such as tendon thickening and altered echogenicity [1,5]. MRI offers superior soft-tissue contrast and allows direct visualization of Achilles tendinopathy, retrocalcaneal bursitis, and bone marrow edema within the calcaneal prominence [1,5]. Recent imaging studies have confirmed the usefulness of MRI for assessing disease severity and detecting associated inflammatory changes [6].

An important aspect of the diagnostic evaluation of posterior heel pain is considering the differential diagnosis. Inflammatory arthropathies, particularly spondyloarthropathies, may present with Achilles tendon enthesitis that can mimic the imaging features of Haglund syndrome. MRI studies have demonstrated that patients with psoriatic arthritis, psoriasis, or ankylosing spondylitis may exhibit subclinical enthesitis of the Achilles tendon and plantar fascia [9].

Despite some overlapping imaging findings, several features may help distinguish inflammatory enthesitis from mechanical tendinopathy. Enthesitis associated with spondyloarthropathies typically presents with more diffuse bone marrow edema, cortical erosions at the calcaneal insertion, and involvement of multiple entheseal sites. Biological workup, including inflammatory markers such as C-reactive protein and erythrocyte sedimentation rate, as well as HLA-B27 status, may provide additional support for an underlying spondyloarthropathy when suspected [9]. In contrast, Haglund deformity is primarily related to mechanical impingement. It is characterized by a prominent posterosuperior calcaneal tuberosity impinging on the Achilles tendon insertion, resulting in stress and degenerative lesions and possible retrocalcaneal bursitis [7]. In our patient, laboratory workup did not reveal evidence of systemic inflammation, further supporting a mechanical rather than an inflammatory process.

Beyond inflammatory enthesitis associated with spondyloarthropathies, several other conditions must be considered in the differential diagnosis of posterior heel pain and can generally be distinguished from Haglund syndrome on imaging. Isolated insertional Achilles tendinopathy, in the absence of an underlying posterosuperior calcaneal prominence, may present with a similar clinical picture of pain and swelling at the tendon insertion; however, imaging in this setting demonstrates tendon thickening and signal alteration without the characteristic bony prominence or the associated retrocalcaneal bursitis that defines Haglund syndrome [1,4]. Isolated retrocalcaneal bursitis, occurring without a prominent calcaneal tuberosity, may also mimic the clinical presentation but lacks the osseous impingement component and is not typically accompanied by adjacent Achilles tendinopathy [1,5]. Calcaneal stress fractures represent a less common but important differential diagnosis, particularly in patients with a recent increase in weight-bearing activity; conventional radiography and, when necessary, MRI or CT typically reveal a linear fracture line surrounded by bone marrow edema, without the characteristic posterosuperior osseous prominence seen in Haglund deformity [1]. Finally, posterior ankle impingement syndromes, such as os trigonum syndrome, may also cause posterior ankle pain but arise from osseous or soft-tissue conflict at the posterior talar process rather than at the calcaneal insertion of the Achilles tendon, and can be differentiated by the anatomical location of the abnormality on imaging [4]. A systematic imaging evaluation, combining radiography with MRI when indicated, therefore allows accurate differentiation of Haglund syndrome from these other causes of posterior heel pain.

Initial management of Haglund syndrome is generally conservative and includes activity modification, physiotherapy, and footwear adaptation. This was sufficient to relieve pain and improve ankle mobility for our patient. Surgical resection of the calcaneal prominence and bursectomy may be considered when symptoms persist despite conservative treatment [10].

## Conclusions

Haglund deformity represents an important mechanical cause of posterior heel pain and may sometimes be visible on physical examination. Although conventional radiography can identify the characteristic posterosuperior calcaneal prominence and enlargement of the posterior aspect of the calcaneus, Haglund syndrome is typically associated with retrocalcaneal bursitis and Achilles tendinopathy, which are only visible on additional imaging modalities such as ultrasound and MRI. The latter provides superior soft-tissue contrast and is the most accurate technique for distinguishing Haglund deformity from inflammatory conditions, such as Achilles enthesitis associated with spondyloarthropathies. The role of CT imaging is secondary and generally reserved for detailed assessment of osseous anatomy or preoperative planning in patients with persistent symptoms despite adequate conservative management.
